# Risk of Pneumonia in Pediatric Patients Following Minor Chest Trauma: A Population-Based Retrospective Cohort Study

**DOI:** 10.3390/ijerph18094690

**Published:** 2021-04-28

**Authors:** Ying-Hsiang Chou, Li-Hsiu Tai, Chi-Ho Chan, Haw-Yu Liu, Han-Wei Yeh, Yu-Hsun Wang, Chiao-Wen Lin, Shun-Fa Yang, Ying-Cheng Chen, Chao-Bin Yeh

**Affiliations:** 1 Department of Radiation Oncology, Chung Shan Medical University Hospital, Taichung 402, Taiwan; hideka.chou@gmail.com; 2 Department of Medical Imaging and Radiological Sciences, Chung Shan Medical University, Taichung 402, Taiwan; 3 Department of Emergency Medicine, School of Medicine, Chung Shan Medical University, Taichung 402, Taiwan; a22leo22@gmail.com (L.-H.T.); 1017gg@gmail.com (H.-Y.L.); 4 Department of Emergency Medicine, Chung Shan Medical University Hospital, Taichung 402, Taiwan; 5 Department of Microbiology and Immunology, Chung Shan Medical University, Taichung 402, Taiwan; chiho@csmu.edu.tw; 6 School of Medicine, Chang Gung University, Taoyuan 333, Taiwan; george66889@gmail.com; 7 Department of Medical Research, Chung Shan Medical University Hospital, Taichung 402, Taiwan; cshe731@csh.org.tw (Y.-H.W.); ysf@csmu.edu.tw (S.-F.Y.); 8 Institute of Oral Sciences, Chung Shan Medical University, Taichung 402, Taiwan; cwlin@csmu.edu.tw; 9 Institute of Medicine, Chung Shan Medical University, Taichung 402, Taiwan; 10 Department of Surgery, Changhua Christian Hospital, Changhua 500, Taiwan

**Keywords:** minor chest trauma, pediatric patients, pneumonia

## Abstract

This study investigated the association between minor chest trauma and the risk of pneumonia among pediatric patients in a Taiwanese health care setting. For this retrospective population-based cohort study, the Longitudinal Health Insurance Database was used to analyze the data of patients with a minor chest injury between 2010 and 2012. Data were analyzed through a multivariate analysis with a multiple Cox regression model. Patients were divided into a chest trauma group (*n* = 6592) and a non-chest trauma group (*n* = 882,623). An increased risk of pneumonia was observed in the chest trauma group (hazard ratio = 1.23; 95% confidence interval = 1.02–1.49) compared to the non-chest trauma group. In conclusion, this population-based cohort study demonstrated that pediatric patients with minor chest trauma are at an increased risk of pneumonia. The short-term adverse effects of pneumonia could be severe when a patient suffers from mild chest trauma.

## 1. Introduction

Chest trauma is a common complaint for emergency department visits. In the United States [1], chest trauma causes approximately 796,000 emergency department visits annually [2] and caused 18,856 hospitalizations during 2002–2004 in Taiwan [3]. In all trauma cases, thoracic injury contributes to 15% of these patients [4,5] and rises to 25% in trauma caused fatalities [6]. Thoracic trauma may cause laceration to chest wall, ribs, lung, heart, great vessels, and tracheobronchial tree. Motor vehicle accidents, fall accidents, and assault injuries are the main causes. There are two mechanisms of thoracic trauma: blunt and penetrating trauma. In the pediatric population, thoracic trauma is less common than in adults due to the greater elasticity and flexibility of the thoracic cage in children. Similarly to adults, traffic accidents are the main causes of mortality and morbidity for children older than one year old with an approximate 5% mortality rate [4,5,7]. Compared to other classes of injury, thoracic injury has been found to have a higher mortality rate of 7.74%, and blunt trauma is the most frequent cause [8]. Pulmonary contusion, rib fractures, pneumothorax, and hemothorax are the most common thoracic injury in the pediatric group [9,10]. Patients with minor thoracic injuries, such as chest abrasion, chest contusion, and closed fractures of the ribs, sternum, or clavicle might be directly discharged after primary management in the emergency department with adequate pain control. However, complications such as pneumonia, empyema, and acute respiratory distress syndrome (ARDS) may occur after chest trauma [6,11,12,13]. About one-third of patients with thoracic trauma develop pulmonary complications such as pneumonia and empyema [6]. Patients with minor thoracic injuries may exhibit pain for several months [14]. This pain restricts the movement of chest wall and leads to hypoventilation. Moreover, this prolonged pain may cause the impairment of the ability to cough, which affects the efficiency of clearing secretions in the airway and results in atelectasis, the accumulation of secretions, and infections [15,16]. Therefore, adequate pain control is important for patients with thoracic trauma.

The incidence of pneumonia following chest trauma was reported to be 1.5% in adult patients [17]. In the pediatric group, pneumonia has been found to be the leading cause of death from infectious disease. In 2013, pneumonia caused approximately 900,000 of the estimated 6.3 million child deaths globally [18]. A previous study showed that elderly patients with rib fractures have a high incidence of pneumonia following chest trauma [19]. Recently, a study showed that an increased number of rib fractures was one of the risk factors for the development of pneumonia [20]. However, no study has investigated the relationship between the incidences of pneumonia following minor thoracic trauma in the pediatric group. Clarifying the relationship between minor blunt chest trauma and delayed pneumonia in the pediatric group is crucial. In this study, we verified whether minor blunt chest trauma increases the risk of pneumonia.

## 2. Materials and Methods

### 2.1. Data Sources

The National Health Insurance Research Database (NHIRD) that was developed by Taiwan’s National Health Research Institutes contains information on the entire population’s medical claims, including diagnoses, medications, procedures, and medical expenditures. NHIRD enrolled almost 99% of the population of 23 million beneficiaries in Taiwan. The Longitudinal Health Insurance Database (LHID) comprises a random sample of the data of 1 million beneficiaries from the NHIRD with no significant difference in age and sex. We collected data from 2009 to 2013. The Ethical Review Board of the Chung Shan Medical University Hospital (CS18096) approved our study.

### 2.2. Study Group

This study used a retrospective cohort design. Participants aged ≤18 years who had experienced chest trauma (ICD-9-CM = 807.0, 807.2, 810.0, 861.2, and 922.0) from 2010 to 2012 were included in our study. The index date was defined as the first date of chest trauma. To avoid confounding with a historic disease, we excluded those with a diagnosis of pneumonia before the index date

**Figure 1:**
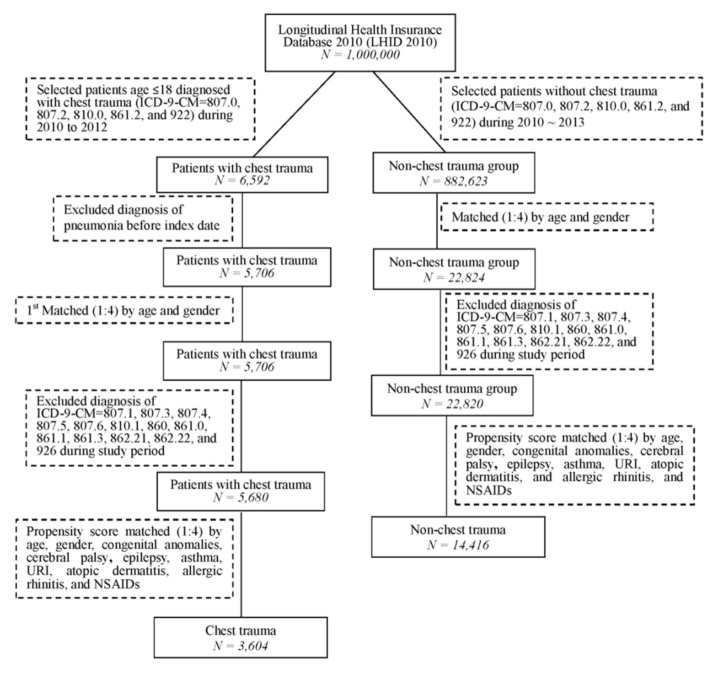
Flowchart for patient selection.

The comparison group consisted of those without chest trauma (ICD-9-CM = 807.0, 807.2, 810.0, 861.2, and 922.0) during 2010–2013. We first matched controls for age and sex at a ratio of 1:4 to obtain an index date corresponding to that of the chest trauma group. We again excluded patients who received a diagnosis of pneumonia before the index date from the non-chest trauma group. To reduce the confounder of a second chest trauma, we excluded patients who received a diagnosis of chest trauma (ICD-9-CM = 807.1, 807.3, 807.4, 807.5, 807.6, 810.1, 860, 861.0, 861.1, 861.3, 862.21, 862.22, and 926) during the study period. Second, a 1:4 propensity-score matching was performed for the non-chest trauma group according to age, sex, congenital anomalies (ICD-9-CM = 740–759), cerebral palsy (ICD-9-CM = 343), epilepsy (ICD-9-CM = 345), asthma (ICD-9-CM = 493), upper respiratory tract infections (ICD-9-CM = 460–466), atopic dermatitis (ICD-9-CM = 691), and allergic rhinitis (ICD-9-CM = 472, 473, and 477). These comorbidities were defined before the index date within 1 year. In addition, the usage of NSAIDs (non-steroid anti-inflammatory drugs) during the study period was included. In total, 3604 patients with minor chest trauma and 14,416 patients without chest trauma were selected for the final analysis. Figure 1 illustrates the study framework.

The outcome was the occurrence of pneumonia (ICD-9-CM = 481, 482, 483, 485, and 486) within 1 year of the index date. All participants were traced until the occurrence of pneumonia, withdrawal from the social insurance system, or 1 year after the index date—whichever occurred first.

### 2.3. Statistical Analysis

The variables in the chest trauma and non-chest trauma groups were compared using the chi-squared test, Fisher’s exact test, or Student’s *t* test, as appropriate. The cumulative incidence of pneumonia was analyzed using the Kaplan–Meier method, and significance was calculated using the log-rank test. The Cox proportional-hazard model was used to estimate the hazard ratios of chest trauma. All statistical analyses were performed using SPSS V.18.0 (SPSS, Chicago, IL, USA). A *p* value of <0.05 was considered statistically significant.

## 3. Results

### 3.1. Characteristics of Study Patients

The characteristics of the patient’s selection are listed in Table 1. The age of most patients in this study was between 12 and 18 years. The mean ages were 12.03 years (SD: 4.37) and 12.15 years (SD: 4.34) in the chest trauma and non-chest trauma groups, respectively. The differences in baseline characteristics between the groups were not significant.

### 3.2. Risk of Pneumonia After Exposure to Chest Trauma

**Figure 2:**
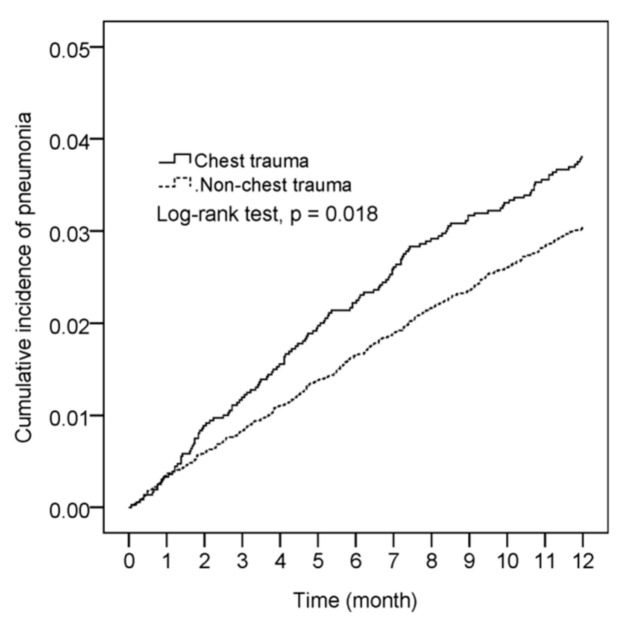
Kaplan–Meier curves of the cumulative proportion of pneumonia.

The Kaplan–Meier plot indicated that children with minor chest trauma had a higher risk of developing pneumonia within one year than those without chest trauma (log-rank test, *p* = 0.018; Figure 2). The Cox proportional-hazard model demonstrated that the patients who had minor chest trauma had a higher risk of subsequent pneumonia (adjusted hazard ratio (aHR) = 1.23; 95% confidence interval (CI) = 1.02–1.49; *p* = 0.034) after adjustment for age, sex, congenital anomalies, epilepsy, asthma, upper respiratory tract infection, atopic dermatitis, allergic rhinitis, and NSAIDs. Furthermore, congenital anomalies (aHR = 1.76; 95% CI = 1.07–2.91; *p* = 0.026), epilepsy (aHR = 4.95; 95% CI = 2.21–11.10; *p* < 0.001), upper respiratory tract infection (aHR = 1.77; 95% CI = 1.27–2.47; *p* = 0.001), and allergic rhinitis (aHR = 1.31; 95% CI = 1.08–1.59; *p* = 0.005) were found to be risk factors of pneumonia in children (Table 2).

### 3.3. Risk of Pneumonia among Patients with and without Chest Trauma and Subgroup-Specific Characteristics

The subgroup analysis demonstrated that the patients aged 12–18 years with minor chest trauma were at a higher risk of pneumonia (HR = 1.48; 95% CI = 1.01–2.18; *p* = 0.046) (Table 3) than those in the non-chest trauma group. The risk of pneumonia was significantly higher in the chest trauma group than in the non-chest trauma group at follow-ups of ≤3 months (aHR = 1.42; 95% CI = 1.00–2.01; *p* = 0.049) and ≤6 months (aHR = 1.33; 95% CI = 1.03–1.71; *p* = 0.028; Table 4).

## 4. Discussion

According to the abbreviated injury score (AIS < 3), minor chest trauma is always the only injury needed symptomatic treatment [21]. In our study, the incidence of pneumonia following minor thoracic injury in pediatric patients was 3.29%. Régulo et al. reported the incidence of pneumonia following chest trauma to be 1.5% [17]. However, they only included patients aged >16 years. Chauny et al. reported an incidence rate of 0.6% for delayed pneumonia in patients older than 65 years [2]. Compared with both studies, our study demonstrated that the pediatric group had a higher risk of pneumonia than the older group following a minor thoracic injury. The possible pathophysiology for developing pneumonia after minor thoracic injury has been discussed in other studies. Through coughing, the human body can remove mucus in the respiratory tract and is a major rescue mechanism of clearance of intrapulmonary mucus [22]. Therefore, coughing is a good reaction for humans. However, patients with chest trauma may experience impaired coughing and secretion clearance because of pain, leading to atelectasis and subsequent pneumonia [14]. The management of blunt thoracic trauma includes adequate pain control, respiratory support, fluid resuscitation, and early mobilization [23,24]. Most patients with blunt thoracic injury can be treated with simple methods, such as an appropriate airway, oxygen support, volume support, and tube thoracotomy, and only 10% of patient required surgical intervention [11]. Pain contributes to much of morbidity, so adequate pain control is crucial for these patients; for adults, epidural analgesia and combinations with different classes of analgesics or opioids alone are recommended [25]. Using methods such as epidural analgesia or intercostal nerve blocks may improve respiratory function among patients with chest injury [26,27]. In one meta-analysis, older adults demonstrated a lower threshold for pain than younger adults. Therefore, age may be a risk factor for delayed pneumonia following minor thoracic trauma [19]. Bulger et al. also demonstrated that of 464 patients with rib fractures, pneumonia occurred in 31% and 17% of elderly and young patients, respectively [28]. In our study, we observed that the patients aged 12–18 years in the chest trauma group had a higher risk of pneumonia after a minor thoracic injury than those in the non-chest trauma group.

In children and adolescents, younger children have been reported to exhibit lower tolerance to pain compared with older children and adolescents [19], which was in contrast with our results that younger children should have a higher incidence of delayed pneumonia. A possible reason of the higher incidence of pneumonia following a minor thoracic injury in children compared with that in adults may be that the pain in children is underestimated and undertreated, especially in very young pediatric patients [29,30,31]. Therefore, evaluating pain in pediatric patients is crucial. We also observed that patients with congenital anomalies, epilepsy, upper respiratory tract infection, and allergic rhinitis are at a higher risk of pneumonia than those without these comorbidities. Teepe et al. included 107 children with either radiologically or clinically diagnosed community-acquired pneumonia during 1999–2008. In these adjusted analyses, patients who were younger, had a history of asthma, and had several previous visits for upper respiratory tract infections were independently associated with a high risk of community-acquired pneumonia [32]. In a different study, the author investigated 201 patients with pneumonia between three months and 15 years of age and demonstrated that histories of recurrent respiratory infections during the past year and wheezing episodes, otitis media, and tympanocentesis before the age of two years were risk factors for community-acquired pneumonia. Among children under five years and in the group of 5–14 years of age, the risk factors were a history of recurrent respiratory infections during the previous year and wheezing periods at any age [33]. During the follow-up, our study results demonstrated that the incidence of pneumonia within six months of trauma was higher in the chest trauma group than in the non-chest trauma group. Chauny et al. reported that only 0.6% of patients developed delayed pneumonia after rib fractures, and no other delayed pneumonia was recorded after 4 and 12 weeks of the fracture [2]. However, this study only included patients older than 65 years and with rib fractures. The administration of a potent NSAID, ketorolac, in rib fracture patients to relief pain and to reduce inflammation would decrease the risk of pneumonia [34]. This finding supports our claim that chest pain might influence effective coughing and increase the risk of pneumonia development.

The strength of this cohort study is the use of a nationwide database, LHID 2010, which includes the data of one million insured people who were randomly selected from the 2010 registry of beneficiaries. The database provides accurate data of medical conditions in Taiwan. However, our study also had several limitations. First, we could only obtain the data of patients with a diagnosis of minor thoracic injury from the datasets. The cause of the injury and detailed clinical information, such as injury severity score (ISS), are not available in the NHIRD. Injury severity was reported to be a risk factor for posttraumatic pneumonia [35]. Antonelli et al. demonstrated that age >40 years and elevated abbreviated injury scale (AIS) scores in thoracic and abdominal trauma were independently associated risk factors for pneumonia within the first four days after trauma, whereas AIS scores of ≥4 for abdominal trauma and exposure to mechanical ventilation for more than five days were associated with the development of pneumonia after four days of trauma occurrence. Second, most patients we enrolled in this study were aged between 12 and 18 years (65.9%), which might have caused selection bias. Third, deposition practice might differ in different countries and emergency departments. Fourth, due to annual restrictions on research funding and available data, we could only obtain data from 2009 to 2013.

## 5. Conclusions

The incidence of pneumonia following minor thoracic injury was 3.29% in pediatric patients, as per our study results. In addition, congenital anomalies, epilepsy, upper respiratory tract infections, and allergic rhinitis are risk factors of pneumonia. Moreover, pediatric patients develop subsequent pneumonia within six months of minor thoracic trauma. Clinicians should be aware of this finding and consider it when treating pediatric patients for minor thoracic trauma.

## Figures and Tables

**Table 1:** Demographic data of study population of chest trauma and non-chest trauma.

Variables	Chest Trauma *N* = 3604		Non-Chest Trauma *N* = 14,416		*p*-Value ^†^
	*n*	%	*n*	%	
Age on index date					0.123
0–5	412	11.4	1563	10.8	
6–11	1203	33.4	4626	32.1	
12–18	1989	55.2	8227	57.1	
Mean ± SD	12.03 ± 4.37		12.15 ± 4.34		0.141
Gender					0.798
Female	1543	42.8	6138	42.6	
Male	2061	57.2	8278	57.4	
Congenital anomalies	44	1.2	223	1.5	0.147
Cerebral palsy	3	0.1	10	0.1	0.781 ^¶^
Epilepsy	15	0.4	38	0.3	0.130
Asthma	116	3.2	475	3.3	0.818
Upper respiratory tract infection	2929	81.3	11,619	80.6	0.360
Atopic dermatitis	100	2.8	468	3.2	0.147
Allergic rhinitis	937	26.0	3720	25.8	0.812
NSAIDs	2186	60.7	8563	59.4	0.169
Track duration (years): mean ± SD	0.98 ± 0.12		0.98 ± 0.11		0.012

^†^ Statistic used chi-squared test or independent *t* test as appropriate. ^¶^ Fisher’s exact test.

**Table 2:** Cox proportional hazard model of pneumonia event.

Variables	No. of Subjects	No. of Pneumonia Event	Crude HR	95% C.I.	*p*-Value	Adjusted HR ^†^	95% C.I.	*p*-Value
Group								
Non-chest trauma	14,416	437	1			1		
Chest trauma	3604	137	1.26	1.04–1.53	0.018	1.23	1.02–1.49	0.034
Age on index date								
0–5	1975	248	1			1		
6–11	5829	193	0.25	0.21–0.30	<0.001	0.26	0.21–0.31	<0.001
12–18	10,216	133	0.10	0.08–0.12	<0.001	0.11	0.08–0.13	<0.001
Gender								
Female	7681	254	1			1		
Male	10,339	320	0.94	0.79–1.10	0.436	1.04	0.88–1.23	0.622
Congenital anomalies	267	16	1.95	1.18–3.20	0.009	1.76	1.07–2.91	0.026
Epilepsy	53	6	3.73	1.67–8.33	0.001	4.95	2.21–11.10	<0.001
Asthma	591	39	2.20	1.59–3.04	<0.001	1.26	0.90–1.75	0.172
Upper respiratory tract infection	14,548	534	3.23	2.34–4.45	<0.001	1.77	1.27–2.47	<0.001
Atopic dermatitis	568	41	2.41	1.75–3.31	<0.001	0.95	0.68–1.31	0.741
Allergic rhinitis	4657	159	1.10	0.92–1.32	0.299	1.31	1.08–1.59	0.005
NSAIDs	10,749	406	1.64	1.37–1.96	<0.001	1.09	0.90–1.32	0.363

^†^ Adjusted for age, gender, congenital anomalies, epilepsy, asthma, upper respiratory tract infection, atopic dermatitis, allergic rhinitis, and NSAIDs.

**Table 3:** Subgroup analysis of Cox proportional hazard model of pneumonia event.

Variables	Chest Trauma		Non-Chest Trauma		HR	95% CI	*p*-Value
	*N*	No. of Pneumonia Event	*N*	No. of Pneumonia Event			
Age							
0–5	412	60	1563	188	1.24	0.93–1.66	0.149
6–11	1203	42	4626	151	1.07	0.76–1.51	0.690
12–18	1989	35	8227	98	1.48	1.01–2.18	0.046
*p* for interaction = 0.468							
Gender							
Female	1543	65	6138	189	1.38	1.04–1.83	0.026
Male	2061	72	8278	248	1.17	0.90–1.52	0.238
*p* for interaction = 0.409							
Congenital anomalies							
No	3560	135	14,193	423	1.28	1.05–1.55	0.013
Yes	44	2	223	14	0.71	0.16–3.14	0.654
*p* for interaction= 0.448							
Epilepsy							
No	3589	136	14,378	432	1.27	1.05–1.54	0.016
Yes	15	1	38	5	0.48	0.06–4.11	0.503
*p* for interaction = 0.375							
Asthma							
No	3488	132	13,941	403	1.32	1.08–1.60	0.006
Yes	116	5	475	34	0.59	0.23–1.52	0.276
*p* for interaction = 0.103							
Upper respiratory tract infection							
No	675	9	2797	31	1.20	0.57–2.52	0.632
Yes	2929	128	11,619	406	1.26	1.03–1.54	0.023
*p* for interaction = 0.900							
Atopic dermatitis							
No	3504	126	13,948	407	1.24	1.01–1.51	0.036
Yes	100	11	468	30	1.77	0.88–3.52	0.107
*p* for interaction = 0.336							
Allergic rhinitis							
No	2667	93	10,696	322	1.16	0.92–1.47	0.197
Yes	937	44	3720	115	1.53	1.08–2.17	0.016
*p* for interaction = 0.198							
NSAIDs							
No	1418	46	5853	122	1.57	1.12–2.20	0.009
Yes	2186	91	8563	315	1.14	0.90–1.43	0.285
*p* for interaction = 0.123							

**Table 4:** Sensitivity analysis for the follow up duration of pneumonia event.

Variables	No. of Subjects	No. of Pneumonia Event	Crude HR	95% C.I.	*p*-Value	Adjusted HR ^†^	95% C.I.	*p*-Value
Follow-up duration ≤1 months								
Group								
Non-chest trauma	14,416	50	1			1		
Chest trauma	3604	12	0.96	0.51–1.80	0.900	0.92	0.49–1.73	0.801
Follow-up duration ≤3 months								
Group								
Non-chest trauma	14,416	118	1			1		
Chest trauma	3604	43	1.46	1.03–2.07	0.034	1.42	1.00–2.01	0.049
Follow-up duration ≤6 months								
Group								
Non-chest trauma	14,416	238	1			1		
Chest trauma	3604	80	1.35	1.05–1.74	0.020	1.33	1.03–1.71	0.028

^†^ Adjusted for age, gender, congenital anomalies, epilepsy, asthma, upper respiratory tract infection, atopic dermatitis, allergic rhinitis, and NSAIDs.

## Data Availability

Restrictions apply to the availability of these data. Data was obtained from National Health Insurance database and are available from the authors with the permission of National Health Insurance Administration of Taiwan.
